# Optimization of the extraction of natural antioxidants from *Coffea robusta* leaves and evaluation of their ability to preserve palm olein from oxidation during accelerated storage

**DOI:** 10.1002/fsn3.702

**Published:** 2018-08-06

**Authors:** Gires Boungo Teboukeu, Fabrice Tonfack Djikeng, Mathilde Julie Klang, Mallampalli Sri Lakshmi Karuna, Hilaire Macaire Womeni

**Affiliations:** ^1^ Department of Biochemistry Faculty of Science University of Dschang Dschang Cameroon; ^2^ School of Agriculture and Natural Resources Catholic University Institute of Buea Buea Cameroon; ^3^ CSIR‐Indian Institute of Chemical Technology Centre for Lipid Research Tarnaka Hyderabad India

**Keywords:** *Coffea robusta*, optimized extract, palm olein, response surface methodology, storage

## Abstract

Response surface methodology (RSM) was used to optimize the extraction of phenolic antioxidants of *Coffea robusta* leaves and to evaluate the effect of optimized extract and storage time on the stability of palm olein. The optimization of the extraction process was conducted, and the total polyphenol value of 127.06 mg GAE/g and scavenging activity of 90.65% were obtained under optimal extraction conditions. The phenolic antioxidants of the optimized extract and their thermal stability were determined using HPLC‐DAD (high‐performance liquid chromatography‐diode array detector) and Rancimat test, respectively. The effect of concentration of the optimized extract and storage time on the stability of palm olein was also evaluated. Results showed that the optimized extract contains gallic acid, vanillic acid, cafeic acid and was efficient in retarding palm olein oxidation during 32 months at room temperature. *Coffea robusta* can be recommended as good source of antioxidants for stabilization of palm olein.

## 1 INTRODUCTION

Oils or fats undergo many transformations and reactions during processing and storage. These changes are favored by many factors including polyunsaturated fatty acid composition, heat, light, oxygen contact, and moisture (Choe & Min, 2006). Autooxidation is the major cause of oil deterioration during storage. During this process, lipid alkyl radicals are formed and react with oxygen to form hydroperoxides which are broken down into secondary oxidation products (Choe & Min, 2006). These oxidation products have been demonstrated as related to mutations, cancers, and cardiovascular diseases (Kubow, 1992; Mc Clements & Decker, 2000).

Synthetic antioxidants such as butylated hydroxytoluene (BHT), butylated hydroxyanisole (BHA), and tertiary butyl hydroquinone (TBHQ) are usually used as ingredient in the food processing sectors to retard oxidation (Allen & Hamilton, 1994). Their role is to inhibit the development of oxidative rancidity in oils. Health issues about the toxicity of synthetic antioxidants present in foods are forcing the food industry to replace these additives with natural ones that are perceived to be “safer” (Krishnaiah, Sarbatly, & Nithyanandam, 2010). Therefore, investigation of natural products has been a major research interest in screening plant materials for possible antioxidant potential. Studies have shown that antioxidant activity of plant extracts is mainly contributed by their phenolic compounds (Djikeng et al., 2017; Jorge, Heliodoro De La Garza, Alejandro, Ruth, & Noé, 2013; Womeni, Tonfack, Anjaneyulu, et al., 2016). However, extraction conditions might affect the amount of phenolic antioxidant extracted. The choice of extraction solvents is critical for complex matrices because the physicochemical properties of the solvents, particularly its polarity, exert an influence on the yields and types of phenolic compound extracted (Balasundram, Sundram, & Samman, 2006). In addition to the solvents used, other factors such as temperature and extraction time also affect the optimization of extraction yield (Luthria, Mukhopadhyay, & Kwansa, 2006; Tomsone, Kruma, & Galoburda, 2012). Moreover, during storage at ambient temperature, the concentration of the extract and storage time can influence the capacity of the extract to retard lipids oxidation (Maleki, Ariaii, & Fallah, 2016; Womeni, Tonfack, Anjaneyulu, et al., 2016).

The objective of this research was to optimize the extraction process of phenolic antioxidants in coffee leaves and to evaluate the effect of optimized extract and storage time on the oxidative stability of palm olein during storage. Palm olein was used in this study because it is the most produced and consumed refined oil in Cameroon. Response surface methodology (RSM) was used for the process optimization. This method establishes a multivariable mathematic model to obtain the relationship between responses and independent variables (Goupy & Creighton, 2006) with the use of a minimal number of experiments.

## 2 MATERIAL AND METHODS

### 2.1 Materials

Refined, bleached, and deodorized palm olein (RBD Palm olein), without additives, was obtained from SCS/RAFCA Palm Oil Industry Company Ltd, Bafoussam, West‐Cameroon. *Coffea robusta* leaves were harvested at Bafang, Haut‐Nkam Division, West‐Cameroon, in January 2015. All the chemicals and reagents used were of analytical reagent grade.

### 2.2 Methods

#### 2.2.1 Optimization of extraction process condition

##### Experimental design

The optimum extraction conditions of polyphenols from *Coffea robusta* leaves were evaluated by Central Composite Design (CCD). The real and coded levels of the various parameters used are shown in Table 1. The intervals of these independent variables were determined on the basis of preliminary studies. A total of 16 experimental runs with two replications at the center point were completed, and the total polyphenols (mgGAE/g) and antioxidant activity (% inhibition) of coffee leaves expressed as the dependent variables were determined (Table 2). This experimental design generates a second‐degree polynomial model (Y) of the form presented in Equation (1):

(1) $$Y=ax_{1}+bx_{2}+cx_{3}+dx_{1}^{2}+ex_{1}x_{2}+fx_{1}x_{3}+gx_{2}^{2}+hx_{2}x_{3}+ix_{3}^{2}+I$$

**Table 1:** Coded and real levels of independent variables used in the RSM design for the optimization process of extraction

Independent variables	Coded levels				
	‐α (−1.73)	−1	0	+1	+ α (1.73)
	Real levels				
Temperature (°C), X_1_	25	31	40	49	55
Extraction time (hours), X_2_	6	9.35	15	20.35	24
Methanol concentration(%), X_3_	0	20	50	80	100

**Table 2:** Experimental design, observed, and predicted values for optimized extraction of phenolic antioxidant from coffee leaves extract

No.	Temperature (°C)	Time (h)	Methanol (%)	Experimental total polyphenols (mg GAE/g)	Predicted total polyphenols	ExperimentalAntioxidant activity (% inhibition)	Predicted antioxidant activity
	X_1_	X_2_	X_3_	EY_1_	PY_1_	EY_2_	PY_2_
01	0 (40)	0 (15)	0 (50)	102.78 ± 0.45	102.81	71.49 ± 0.70	71.48
02	0 (40)	0 (15)	0 (50)	101.56 ± 1.02	102.81	72.33 ± 1.12	71.36
03	−1 (31)	−1 (9.35)	−1 (20)	72.01 ± 1.78	77.96	51.86 ± 1.01	52.61
04	+1 (49)	−1 (9.35)	−1 (20)	70.01 ± 1.48	74.40	74.82 ± 0.09	75.12
05	−1 (31)	−1 (9.35)	+1 (80)	97.80 ± 1.56	109.78	85.80 ± 0.14	87.14
06	+1 (49)	−1 (9.35)	+1 (80)	108.65 ± 1.98	117.67	89.44 ± 0.78	89.17
07	−1 (31)	+1 (20.35)	−1 (20)	79.97 ± 0.20	76.21	75.88 ± 0.75	74.45
08	+1 (49)	+1 (20.35)	−1 (20)	76.03 ± 0.00	69.31	78.70 ± 0.96	79.04
09	−1 (31)	+1 (20.35)	+1 (80)	106.05 ± 0.75	106.92	81.04 ± 1.15	81.46
10	+1 (49)	+1 (20.35)	+1 (80)	112.15 ± 0.16	111.46	85.09 ± 1.25	85.47
11	− α (25)	0 (15)	0 (50)	109.17 ± 1.88	102.77	70.34 ± 1.14	71.01
12	+ α (55)	0 (15)	0 (50)	104.64 ± 1.46	103.60	73.83 ± 1.45	72.89
13	0 (40)	0 (15)	− α (0)	33.59 ± 1.26	36.20	60.01 ± 1.03	60.78
14	0 (40)	0 (15)	+ α (100)	108.47 ± 0.21	98.41	91.80 ± 2.78	91.72
15	0 (40)	− α (06)	0 (50)	129.54 ± 0.19	113.44	77.86 ± 2.21	77.56
16	0 (40)	+α (24)	0 (50)	98.10 ± 0.48	106.75	68.09 ± 0.78	68.59

where Y represents the response variables (total polyphenols or antioxidant activity); x_1_, *x*_2_, and *x*_3_ are the levels of the independent variables; a, b, and c are the linear terms; e, f, and h are the interaction terms; d, g, and i are the quadratic terms and I is a constant.

To confirm or validate the optimum conditions of polyphenol antioxidants extraction, two experimental replicates were performed under optimized conditions. The experimental and predicted values were compared.

##### Extraction of phenolic compounds from coffee leaves

The extraction procedures were carried out randomly and in accordance with the conditions set by the experimental design. Fresh *Coffea robusta* leaves were dried in an electric oven (Venticell*,* MMM, Einrichtungen, Germany) at 50°C for 48 hr. The dried leaves were grounded to pass through a 1‐mm sieve. Sample powder (7 g) was blended with 150 ml of solvent (Methanol/water) of concentration specified by the full factorial design (Table 2). The mixture was placed in an electric oven (Venticell*,* MMM, Einrichtungen, Germany) and regularly subjected to agitation (400 rpm) at the required temperature and time specified by the experimental design (Table 2). The filtrate was concentrated on a rotary evaporator (*BUCHI*, Pharma and Biotech, Germany) at 45°C before being stored at 4°C for further analysis.

##### Determination of total phenolic compounds (TPC)

The total phenolic of the extracts was evaluated using the Folin‐Ciocalteu colorimetric method as described by Gao, Ohlander, Jeppsson, Björk, and Trajkovski (2000). Briefly, plant extract (20 μl) was added in a test tube containing 2 ml of distilled water and 0.2 ml of Folin‐Ciocalteu reagent and incubated for 3 min at room temperature. One (1) ml of 20% sodium carbonate was added to the mixture and re‐incubated for 20 min at 40°C. The absorbance of the resulting blue color was determined at 765 nm using a spectrophotometer (HELIOS Epsilone, Dreieich, Germany). The standard curve prepared from Gallic acid solution was used to express the results as gallic acid equivalents (GAE) per gram of extract.

##### Determination of antioxidant activity

The DPPH (2.2′‐diphenyl‐1‐picrylhydrazyl) radical scavenging activity was determined according to the modified method of Mensor et al. (2001). Nine hundred (900) μl of 0.3 mM methanolic solution of DPPH was added to 100 μl of the samples containing extract at the concentration of 100 μg/ml. The samples were kept in the dark at room temperature, and after 30 min, the absorbance was determined at 517 nm. The absorbance (Abs) of the samples, the control and the blank, was determined against that of methanol. The results were expressed as percent inhibition using Equation (2):

(2) $$\%\text{Inhibition}=[\left({\text{Abs}}_{\mathrm{control}}-{\text{Abs}}_{\mathrm{sample}}\right)\times 100/{\text{Abs}}_{\mathrm{control}}]$$

#### 2.2.2 Phenolic compounds profile by HPLC‐DAD

Phenolic compounds of coffee leaves were determined in the optimized extract obtained under the optimal extraction conditions defined by the experimental design about TPC. The extract was dissolved in methanol (1 mg/ml). The analysis was carried out in an HPLC Agilent system 1200 series equipped with a quaternary pump model G11311A and Diode Array Detector (DAD) (G11315B, Waldbronn, Germany). Data acquisition was performed using Chemstation software. The column type was an RP‐C18 Lichrospher column, 5 μm, 4.0 mm internal diameter × 250 mm. Separations were performed in the isocratic mode, using acetonitrile‐1% orthophosphoric acid in water (70:30 v/v) at a flow rate of 1 ml/min, with an injection volume of 20 μl (sample and standards solution). Identification of phenolic compounds (DAD detection at 280 nm) in extract was achieved by comparing their retention time with those of standards available.

#### 2.2.3 Rancimat test

Rancimat test is used for the evaluation of the antioxidant potential of molecules to limit oxidation of oils and fats. Oil samples used for this test were prepared according to the modified method of Iqbal, Haleem, Akhtar, Zia‐ul‐Haq, and Akbar (2008). The optimized extract was separately added to preheated RBD palm olein (at 50°C for 3 hr) at concentrations of 500, 720, 1250, 1780, and 2000 ppm. The efficacy of natural antioxidants was evaluated by comparing their antioxidation activity with those of butylatedhydroxytoluene (BHT) employed at it legal limit of 200 ppm (Duh & Yen, 1997). Palm olein without additives and prepared under the same conditions served as control.

Induction periods of stabilized (oil containing the optimized extract) and control oil samples were evaluated using an automated Metrohm Rancimat (model 892, Germany) as described by Womeni, Tonfack, Anjaneyulu, et al. (2016). The time elapsed from the beginning until the oil starts to become rancid (induction period) was automatically recorded by the instrument. The protection factor was calculated using the induction time of oil with antioxidant (*I*) and the induction time of oil without antioxidant (*I*_0_).

$$\text{Protection factor}=I/I_{0}$$

#### 2.2.4 Effect of optimized extract concentration and storage time on the oxidative stability of palm olein during storage

##### Experimental design

The effects of process parameters (concentration of extract and storage time) on the oxidative stability of palm olein were evaluated by Central Composite Design (CCD). Real and coded levels of the independent variables used are shown in Table 3, and the intervals were determined on the basis of other studies (Womeni, Tonfack, Anjaneyulu, et al., 2016; Womeni, Tonfack, Iruku, et al., 2016) and preliminary test. The design consisted of 10 runs with two replicates at the center point, and the peroxide, *p*‐Anisidine, and total oxidation (TOTOX) values assays expressed as the dependent variables were determined (Table 4). A full quadratic model was used for fitting of data (Equation (3)):

(3) $$Y^{\prime }=I+ax_{1}+bx_{2}+cx_{1}^{2}+dx_{2}^{2}+ex_{1}x_{2}$$

**Table 3:** Coded and real levels of independent variables used in the RSM design for study effect of extract concentration and storage time on the oxidative stability of palm olein

Independent variables	Coded levels				
	−α (−1.68)	−1	0	+1	+ α (1.68)
	Real levels				
Extract Concentration (ppm), X_1_	500	720	1250	1780	2000
Storage time (Days), X_2_	0	7	23	39	46

**Table 4:** Experimental design, observed and predicted values of parameter effect on the oxidative stability of palm olein during storage

No.	Extract (ppm)	Storage time (days)	Experimental Peroxide value (ppm)	Predicted Peroxide value (ppm)	Experimental *p*‐Anisidine value	Predicted *p*‐Anisidine value	Experimental TOTOX value	Predicted TOTOX value
	X_1_	X_2_	EY_1_	PY_1_	EY_2_	PY_2_	EY_3_	PY_3_
01	0 (1250)	0 (23)	13.20 ± 0.45	13.20	2.26 ± 0.04	2.26	28.68 ± 0.94	28.68
02	0 (1250)	0 (23)	13.20 ± 1.04	13.20	2.26 ± 0.12	2.26	28.68 ± 2.20	28.68
03	1 (1780)	1 (39)	15.24 ± 0.98	16.99	2.94 ± 0.08	2.80	33.43 ± 2.04	36.78
04	1 (1780)	−1 (7)	3.92 ± 0.99	4.31	2.23 ± 0.06	2.08	10.07 ± 2.04	10.72
05	−1 (720)	1 (39)	23.98 ± 0.12	25.20	3.10 ± 0.19	3.02	51.07 ± 0.43	53.42
06	−1 (720)	−1 (7)	3.66 ± 0.13	3.52	2.58 ± 0.00	2.50	9.91 ± 0.26	9.55
07	α (2000)	0 (23)	9.25 ± 1.46	8.06	2.19 ± 0.26	2.35	20.70 ± 3.70	18.49
08	−α (500)	0 (23)	13.73 ± 1.03	13.31	2.73 ± 0.30	2.80	30.21 ± 2.36	29.42
09	0 (1250)	α (46)	28.23 ± 0.63	26.47	2.95 ± 0.05	3.06	59.42 ± 1.31	56.01
10	0 (1250)	−α (0)	2.03 ± 0.61	2.18	2.08 ± 0.10	2.19	6.14 ± 1.32	6.56

where Y^’^ represents the response variables (peroxide value, *p*‐Anisidine value and TOTOX value); *x*_1_ and *x*_2_ are the levels of the independent variables; *a* and *b* are the linear terms; e is the interaction term; c and d are the quadratic terms and I is a constant.

##### Sample preparation

The optimized extract was dissolved and separately added to 60 g of preheated RBD palm olein (at 50°C for 3 hr) at concentrations indicated by the full factorial design (Table 4). Stabilized oil samples were placed in dark brown glass bottles with narrow necks and subjected to accelerated storage in an electric hot air oven at 70°C (08 h heating cycle per day) for 46 days. Samples were collected according to the time (days) indicated by the experimental design and stored in the refrigerator for further analysis. The oxidative deterioration level was assessed by determining oxidation parameters.

##### Evaluation of oxidation parameters

The peroxide value (PV) of oil samples was determined following the spectrophotometrical IDF standard method, 74A: 1991 (International Dairy federation, 1991). The *p*‐Anisidine value (AV) was assessed according to AOCS Official Method CD 18–90 (AOCS, 1999) and the total oxidation (TOTOX) calculated using the following equation: TOTOX= 2 PV + AV (Shahidi & Wanasundara, 2008).

#### 2.2.5 Statistical analyses

STATGRAPHICS Plus 5.0 was used for the experimental design and statistical analysis of the data. All responses were determined in duplicate, and the power of the model was determined by evaluating the coefficient of determination (*R* ^2^) obtained from the analysis of variance (ANOVA). Statistical significance of the model variables was determined at 5% probability level. Response surfaces and contour plots were plotted using Sigma Plot v11.0 (c) systat.

The Rancimat test was performed in duplicate, and results were represented as means ± standard deviations. The Dunnett and Student‐Newmann‐Keuls tests were used to compare means using the software GraphPad‐InStat, version 3.05 for Windows.

## 3 RESULTS AND DISCUSSION

### 3.1 Optimization of extraction by RSM

#### 3.1.1 Analysis of variance and regression equations

Table 2 shows the results of the antioxidant activity (% Inhibition) and total polyphenols (mgGAE/g) of coffee leaves extract. The higher phenolic content registered in this study can be attributed particularly to the solvent used for extraction (Ghumman, Singh, & Kaur, 2017). In fact, methanolic extracts have been reported as exhibiting highest TPC and antioxidant activity compared to ethanol and acetone (Sulaiman, Sajak, Ooi, Supriatno, & Seow, 2011).

The experimental design has been formulated to develop an empirical model to investigate the interaction of different associated independent variables responsible for the extraction of phenolic antioxidants present in coffee leaves and also, to identify the optimum conditions of extraction. The analysis of variance (ANOVA) presented in Table 5 showed that solvent (methanol/water mixture) is the only factor in the linear terms that significantly affect (*p *< 0.05) the total phenolic content and antioxidant activity. Methanol/water in quadratic term had significant (*p *< 0.05) effect on TPC. Results also showed that the coefficient of determination (*R* ^2^ value) of the responses is within the range of a good set (more than 0.75) (Joglekar & May, 1987), indicating that the model could explain adequately up to 89.5% and 84.9% of TPC and antioxidant activity, respectively. The mathematical models of relationship for total phenolic content (Y_1_) and antioxidant activity (Y_2_) with temperature (X_1_), time (X_2_) and methanol fraction (X_3_) are given by the Equations (4) and (5):

(4) $$\begin{array}{cc} Y_{1}= & 72.71-0.38X_{1}-2.13X_{2}+1.61X_{3}+\\ & 0.001X_{1}^{2}-0.01X_{1}X_{2}+0.01X_{1}X_{3}+0.08X_{2}^{2}-0.001X_{2}X_{3}-0.01X_{3}^{2} \end{array}$$

(5) $$\begin{array}{cc} Y_{2}\;= & 13.4+0.48X_{1}+2.1X_{2}+0.76X_{3}+\\ & 0.01X_{1}^{2}-0.04X_{1}X_{2}-0.008X_{1}X_{3}+0.04X_{2}^{2}-0.02X_{2}X_{3}+0.002X_{3}^{2} \end{array}$$

**Table 5:** Regression coefficients (RC), *p* values, and coefficient of multiple determinations (*R* ^2^) for total phenolic content and antioxidant activity of coffee leaves extract following CCD

	Total polyphenols		Antioxidant activity	
	RC	*p* value	RC	*p* value
X_1 _: Temperature (°C)	−0.3831	0.9405	0.481	0.1449
X_2 _: Time (hours)	−2.1311	0.5541	2.124	0.9231
X_3 _: Methanol fraction (%)	1.6123	0.0011^a^	0.766	0.0029^a^
X_1_X_1_	0.0016	0.9735	0.012	0.645
X_1_ X_2_	0.0168	0.8469	−0.0498	0.314
X_1_ X_3_	0.0105	0.5160	−0.008	0.353
X_2_X_2_	0.0851	0.5286	0.043	0.548
X_2_ X_3_	−0.0016	0.9486	−0.028	0.0852
X_3_X_3_	−0.0139	0.0173^a^	0.0026	0.301
Constant	72.7127		13.40	
*R* ^2^	0.895		0.849	
*R* ^2^ (adjusted)	0.738		0.624	

*Note*

a Independent variable that significantly (*p *<* *0.05) affect the response.

#### 3.1.2 Analysis of response surfaces

**Figure 1:**
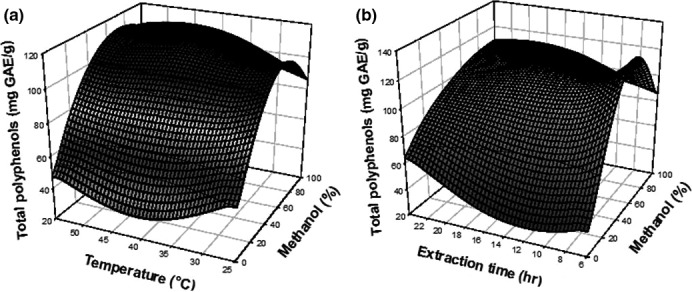
Response surfaces showing the effect of temperature and solvent fraction (a), extraction time and solvent fraction (b) on the total phenolic content of *Coffea robusta* leaves extract

**Figure 2:**
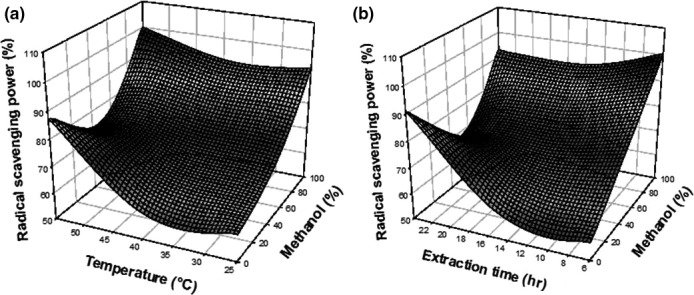
Response surfaces showing the effect of temperature and solvent fraction (a), extraction time and solvent fraction (b) on the antioxidant activity of *Coffea robusta* leaves extract

According to the equation, the effect of the three independent variables on the total phenolic content (Figure 1) and antioxidant activity (Figure 2) was illustrated using response surfaces. The total phenolic content and antioxidant activity were proportionally increasing with the percentage of methanol used during extraction. However, at a specific percentage (methanol 80%), the TPC decreases. The responses were also increasing with extraction temperature. For long extraction time, a decrease in the antioxidant activity was observed. The mixtures water and other polar solvent have been shown to be more effective in extracting polyphenols; water led to the creation of a moderately polar medium (Chirinos, Rogez, Campos, Pedreschi, & Larondelle, 2007; Olivas‐Aguirre et al., 2017). The increase in temperature during extraction process leads to an increase in diffusion phenomena that helps to extract polyphenols present in the plant, due to vibratory effects of the cell wall molecules which facilitates migration of free molecules to the solvent (Moahamad, Ali, & Ahmad, 2010). Long extraction time lead to oxidation and chemical losses of the bioactive compounds by extended exposure to oxygen and light (Gomes & Torres, 2016).

#### 3.1.3 Process optimization and validation of the optimal conditions

Table 6 presents the optimal conditions for each individual response with the experimental and predictive value. The maximal response of phenolic extraction corresponded to conditions: 53.70°C with incubation time of 5.60 hr using methanol fraction of 79.66%. The best conditions for maximal DPPH radical scavenging activities were obtained at 47.70°C with incubation time of 5.60 hr using solvent fraction of 100%. Under these conditions, the highest total polyphenols and antioxidant activity were 127.06 mgGAE/g and 90.65%, respectively. The experimental values for the same process include the total phenolic content and antioxidant activity values of 125.35 ± 3.46 mgGAE/g and 89.12 ± 0.20%, respectively. The predicted and experimental values did not vary significantly at 5% level. This shows that the models obtained can be accepted and used to prepare *Coffea robusta* leaves extract with the best phenolic content and high antioxidant activity.

**Table 6:** Predictive and experimental values under optimum conditions for maximum total phenolic content and antioxidant activity

Responses	Temperature (°C)	Extraction time (hr)	Methanol fraction (%)	Predicted value	Experimental value
Total polyphenols (mg GAE/g)	53.70	5.60	79.66	127.06 ± 0.0^a^	125.35 ± 3.46^a^
Antioxidant activity (% inhibition)	47.70	5.6	100	90.65 ± 0.0^a^	89.12 ± 0.20^a^

*Note*

Means within each row with same superscripts are not significantly (*p *< 0.05) different.

### 3.2 HPLC‐DAD analysis of *Coffea robusta* leaves extract

**Figure 3:**
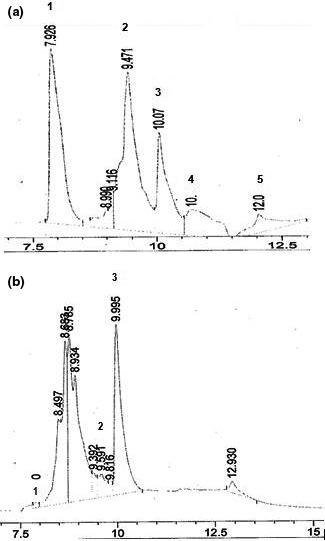
HPLC‐DAD Chromatogram of standards (A) (1 =  Gallic acid, 2 =  Vanillic acid, 3 =  Cafeic acid, 4 =  Ferulic acid, 5 =  Ellagic acid), and *Coffea robusta leaves* extract (B) (1 =  Gallic acid, 2 =  Vanillic acid, 3 =  Cafeic acid)

Phenolic compound profile in coffee leaves extract was determined by HPLC‐DAD. As shown in Figure 3, the peaks 1, 2, and 3 were found to be matching well with gallic acid (retention time: 7.910), vanillic acid (retention time: 9.591), and cafeic acid (retention time: 9.995), respectively. The presence of these phenolic compounds in *Coffea robusta* extract has not been reported in other studies.

### 3.3 Rancimat test for verification of antioxidation activity of *Coffea robusta* leaves extract

The Rancimat test was performed to evaluate the efficiency of the extract in delaying palm olein oxidation. The effects of different concentrations of extract on the protection factors and induction periods of palm olein in comparison with the oil supplemented with BHT (synthetic antioxidant) and control (palm olein free from additives) are presented in Table 7. The addition of the extract at concentrations range 720–2000 ppm significantly (*p *< 0.05) increased the protection factors and prolonged the induction periods (IP) of palm olein compared to the control and palm olein supplemented with BHT. The protection factors and induction periods were increasing with the concentration of the extract. The observed efficiency of *Coffea robusta* leaves extract in prolonging induction time of oil as well as its good protection factors might be attributed to the phenolic antioxidants present in this extract. These results are in accordance with those reported by Womeni, Tonfack, Anjaneyulu, et al. (2016) in the same oil system supplemented with soursop flowers extract at concentrations range 200–1800 ppm.

**Table 7:** Induction times and protection factors of oil samples during storage at 110 °C

Sample	Induction time (hr)	Protection factor
Control	23.19 ± 0.12^a^	1.000 ± 0.00^a^
PO + BHT_200 ppm_	24.41 ± 0.23^b^	1.052 ± 0.007^b^
OP + Cof rob_2000_	27.27 ± 0.51^e^	1.170 ± 0.008^f^
OP + Cof rob_1780_	27.12 ± 0.47^e^	1.169 ± 0.0045^e^
OP + Cof rob_1250_	26.61 ± 0.36^d^	1.147 ± 0.004^d^
OP + Cof rob_720_	25.36 ± 0.29^c^	1.093 ± 0.005^c^
OP + Cof rob_500_	23.79 ± 0.47^ab^	1.025 ± 0.003^b^

*Note*

Values of columns with different letters differ significantly *p *< 0.05. (Control: Palm olein without antioxidant; PO + BHT_200 ppm_: palm olein containing BHT as antioxidant at concentration of 200 ppm; PO + Cof rob_2000 ppm_: palm olein supplemented with *Coffea robusta* extract at concentration 2000 ppm).

### 3.4 Effect of extract concentration and storage time on the oxidative stability of palm olein during storage

The peroxide, *p*‐Anisidine, and total oxidation values of palm olein supplemented with coffee leaves extract obtained from the 10 experiments are presented in Table 4. Concentration of extract and storage time was taken as independent variables. The fresh RBD palm olein free from additives (experiment N°10) was of good quality, as shown by its low peroxide value (<10 ppm), low *p*‐Anisidine value (≤ 20), and low TOTOX value (<26) as recommended by homologation (Codex Alimentarius, 1999, 2015).

#### 3.4.1 Analysis of variance

The experimental data were used to calculate the coefficients of the second‐order polynomial equation, to establish the coefficient of determination (*R* ^2^) and significant effect of independent variables (Table 8). The coefficient of determinations (*R* ^2^) for peroxide, *p*‐Anisidine and TOTOX values being 0.985, 0.886, and 0.987, respectively; and falling within a good range (more than 0.75) (Joglekar & May, 1987). This means that the observed model is able to explain 98.5%, 88.6%, and 98.7% of the results in the case of peroxide value, *p*‐Anisidine value, and TOTOX value, respectively. The analysis of variance (ANOVA) also showed that the two independent variables in interaction or linear terms significantly affects (*p *< 0.05) the peroxide and TOTOX values of oil samples. Only the storage time significantly influence (*p *< 0.05) *p*‐Anisidine value of oils samples during storage.

**Table 8:** Regression coefficients (RC), *p* values, and coefficient of multiple determinations (*R* ^2^) for peroxide value, *p*‐Anisidine value, and TOTOX value following CCD

	Peroxide value		*p*‐Anisidine value		TOTOX value	
	CR	*p* value	CR	*p* value	CR	*p* value
X1 : Extract (ppm)	0.0153	0.028^a^	−0.0026	0.102	0.027	0.020^a^
X2 : Storage time (days)	0.775	0.0001^a^	−0.036	0.014^a^	1.498	0.0001^a^
X1 X1	−0.000005	0.119	8.90^E^‐7	0.066	−0.000008	0.142
X1 X2	−0.0002	0.044^a^	0.000005	0.660	−0.0005	0.039^a^
X2 X2	0.0014	0.625	0.001	0.050	0.0041	0.454
Constant	−9.060		4.133		−13.437	
*R* ^2^ (%)	98.53	88.68	98.72			
*R* ^2^ (adjusted) (%)	96.71	76.26	97.12			

*Note*

a Independent variable that significantly (*p *< 0.05) affect the response.

The mathematical expression of relationship for peroxide value (Y_3_), *p*‐Anisidine value (Y_4_) and TOTOX value (Y_5_) with independent variables are given in the Equations (6), (7) and (8):

(6) $$Y_{3}=-9.06+0.015X_{1}+0.77X_{2}-0.000005X_{1}^{2}-0.0002X_{1}X_{2}+0.001X_{2}^{2}$$

(7) $$Y_{4}=4.13-0.002X_{1}-0.03X_{2}+8.90223E-7X_{1}^{2}+0.000005X_{1}X_{2}+0.001X_{2}^{2}$$

(8) $$Y_{5}=-13.43+0.02X_{1}+1.49X_{2}-0.000008X_{1}^{2}-0.0005X_{1}X_{2}+0.004X_{2}^{2}$$

#### 3.4.2 Analysis of contour plots

**Figure 4:**
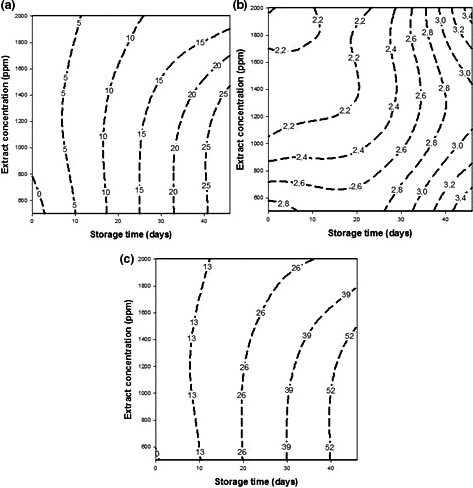
Contour plots showing the effect of storage time and extract concentration on the peroxide value (a), *p*‐Anisidine value (b) and TOTOX value (c) of palm olein during storage

The contour plots showing the effect of the storage time and extract concentration on the peroxide value (a), *p*‐Anisidine value (b), and TOTOX value(c) of oil samples containing *Coffea robusta* leaves extract during storage are presented in Figure 4.

Peroxide value (PV) is a good indicator of the extent of primary oxidation products in oil (Anwar, Siddiq, Iqbal, & Asi, 2007). It measures hydroperoxides of oils and according to the Codex Alimentarius (1999), and palm olein is generally recognized as safe if the PV < 10 ppm. As presented Figure 4a, the peroxide value was proportionally increasing with the storage time. Nevertheless, for high concentrations of extracts, this response increases slightly. The observed efficiency of coffee leaves in prolonging storage time of palm olein might be attributed to the phenolic compounds present in the extract. Phenolic antioxidants are able to donate their hydrogen atom for the stabilization of free radicals present in oil and consequently increase its oxidative stability (Gordon, 1990). According to the Codex alimentarius (1999), it would be advisable to heat at 70°C palm olein enriched with coffee leaves extract at 2000 ppm for 25 days in order to preserve it quality.

The measurement of *p*‐Anisidine value is intensively used to assess secondary oxidation products like 2‐alkenal, 2,4‐alkadienal (Anwar, Qayum, Hussain, & Iqbal, 2010). Contour plot (Figure 4b) shows that these responses increase slightly with the storage time of the oil at 70°C. However, the addition of the extract at high concentrations does not stop the production of secondary oxidation products. In fact, hydroperoxides are thermolabile molecules which easily breakdown into secondary oxidation products at high temperature (>120°C). Codex Alimentarius (2015) recommends a *p*‐Anisidine value lower than 20 for good quality fish oil. Considering this standard, it can be observed that this response was lower than 20 during the entire treatment.

Total oxidation value measures both primary and secondary oxidation products and provides a better determination of the progressive oxidative deterioration of oils (Womeni, Tonfack, Iruku, et al., 2016). Figure 4c illustrates the effect of storage time and extract concentration of *Coffea robusta* leaves on the TOTOX value during the storage. As previously observed with the other dependent variables, the TOTOX value increases with storage time. For high concentrations of extracts, this response increases slightly. The ability of *Coffea robusta* leaves extract to extend the storage time of palm olein might be attributed to their capacity to stabilize the free radicals present in oil; this leads to the inhibition of the formation of hydroperoxides and their breakdown products. Similar results were obtained by Womeni, Tonfack, Iruku, et al. (2016) who demonstrated that extracts of tea leaves significantly retard the total oxidation of palm olein during an accelerated storage of 30 days at 70°C. On the basis of the quality standards for oils with respect to TOTOX value (≤26 according to Codex Alimentarius (2015)), it would be advisable to store at 70°C, palm olein supplemented with coffee leaves extract at 2000 ppm during 32 days in order to preserve its quality.

The Schaal oven test (storage at 65–70°C) is a good simulation of normal storage conditions. Evans, List, Moser, and Cowan (1973) showed that heating of oils for 08 hr at 65°C is equivalent to 1‐month storage at room temperature. During storage of palm olein at 70°C, *Coffea robusta* leaves extract at 2000 ppm has efficiency to preserve its quality during 32 days (08 h per day). Considering assertion of Evans et al. (1973), we can conclude that the supplementation of palm olein with 2000 ppm of *Coffea robusta* leaves extract can extend its preservation to 32 months at room temperature.

## 4 CONCLUSION

Based on the experimental design that had been performed using response surface methodology, the optimal conditions for the extraction of antioxidant phenolic compounds from *Coffea robusta* leaves extract were determined (extraction at 53.70°C with incubation time of 5.60 hr, using methanol fraction of 79.66%). This extract under optimal conditions contains several phenolic compounds and has a good thermal stability. Oxidation of palm olein increases with storage time, and coffee leaves extract have the capacity to delay this alteration reaction and stabilize the oil. This study can be useful for the development of industrial extraction process of coffee leaves and its application as an ingredient to delay lipid oxidation in oils.

## CONFLICT OF INTEREST

None.

## ETHICAL STATEMENT

Humans and animals testing is not applicable to this study.
